# Case of Perforation of Intestine

**Published:** 1852-09

**Authors:** Edwin R. Maxson

**Affiliations:** Adams Centre, Mass.


ART. III.— *Case of Perforation of Intestine.* Reported by EDWIN R. MAXSON, M. D.

Mr. H——, a respectable merchant, aged 57 years, had been troubled for several years with dyspepsia, attended with acid eructations, and vomiting, generally for a week or two, in March and September of each year.

As the difficulty rather increased, he had been induced, by the advice of a quack, to take, for a long time, a composition containing capsicum. This he took daily for near twelve months, at the end of which time he grew much worse. His vomiting became very obstinate. In this condition I was called to see him in March, 1848. I ordered sinapisms over the stomach, and gave Hydg. Mit. gr. ¼ with Pulv. Dov. gr. ½, every two hours. In eight hours the vomiting was checked, so that I gave him Bismuth Sub. Nit., gr. iii, every six hours. This was discontinued at the end of three days, after which I gave him a weak infusion of Columbo. He soon recovered his strength, and his health continued tolerable till December 1st following, when he felt a slight uneasiness in the epigastric region, caused, as he supposed, by lifting. He kept about his business till December 4th, when he was suddenly distressed in the right and lower part of the epigastric region; and being faint he called for brandy, which was administered with little effect.

I saw him in half an hour. He was in great distress, groaning at every breath; the pain occupying the small spot above referred to. I applied a sinapism over the bowels, and gave Tr. Opii, gtt. xxx, with Gum Camph. gr. i, which dose was repeated in one hour, with little or no effect. I then gave Hydg. Mit. gr. xii, in half an ounce of castor oil. This was followed by Castor Oil ℥ss. every two hours; and this by an enema, which produced a slight movement of the bowels. During all this time, he had not breathed without a groan. He had taken a bowl of warm gruel, which gave him considerable pain through the bowels, and there began to be some tenderness, and slight distension of the abdomen.

His pulse had been continually, and was now rapidly sinking. Brandy had no effect; and in twenty hours from the time he was taken he expired. All the symptoms induced me to believe there was perforation of the intestine. This I declared to the friends, and requested an examination, which

I was permitted to make forty-eight hours after death, in the presence of several gentlemen. The cavity of the abdomen contained about two quarts of a darkish fluid. The peritoneum was nearly of the same color; and there was a smooth circular perforation of the duodenum, the size of a half dime.

Adams Centre, Mass., August, 1852.

